# Evaluation of Gross Motor Coordination and Physical Fitness in Children: Comparison between Soccer and Multisport Activities

**DOI:** 10.3390/ijerph17165902

**Published:** 2020-08-14

**Authors:** Boris Popović, Marko Gušić, Danilo Radanović, Slobodan Andrašić, Dejan M. Madić, Draženka Mačak, Dušan Stupar, Goran Đukić, Dragan Grujičić, Nebojša Trajković

**Affiliations:** 1 Faculty of Sport and Physical Education, University of Novi Sad, 21000 Novi Sad, Serbia; borispopovic0803@gmail.com (B.P.); gusicmarko@yahoo.com (M.G.); radanilo17@gmail.com (D.R.); dekimadic@gmail.com (D.M.M.); macak.md@yahoo.com (D.M.); 2 Faculty of Economics, University of Novi Sad, 24000 Subotica, Serbia; andrasicslobodan@yahoo.com; 3 Faculty of Sport and Tourism, Educons University, 21000 Novi Sad, Serbia; dusan.stupar@tims.edu.rs (D.S.); gor5@sbb.rs (G.Đ.); dragan.grujicic@tims.edu.rs (D.G.)

**Keywords:** motor competence, motor abilities, youth, team sport, multisport

## Abstract

The early detection and continuous monitoring of children’s motor competence levels and physical fitness is very important. The purpose of this study was to determine the differences in motor coordination of children enrolled in soccer and multisport activities. The participants of this study included 147 boys and girls (mean age 7.60 ± 0.85 years). The total sample of subjects was composed of two subgroups: children who were enrolled in organized exercise programs—multisports (*n* = 77), and children who were engaged in soccer training (*n* = 70). Motor coordination was evaluated with the Kiphard–Schilling body coordination test (KTK). Physical fitness was assessed with a 20 m shuttle run test, 4 × 10 m shuttle run test, standing long jump, and handgrip strength. The ANCOVA showed significant differences (*p* < 0.05) with large effect size between groups for tests hopping for height (d = 0.93), total motor quotient (d = 1.31), jumping sideways (d = 1.32), and moving sideways (d = 1.59), after adjusting for age and gender. There were no significant differences between groups in the physical fitness tests. It can be concluded that children enrolled in multisport activities have higher levels of motor coordination than children who are enrolled only in soccer. Therefore, multiple sport training programs should be considered and encouraged by parents, educators, and other training professionals.

## 1. Introduction

The effects of different physical activities in the prevention of various diseases (obesity, diabetes, heart disease) in the developmental age, and the promotion of correct lifestyles through multicomponent and interinstitutional interventions at school, sports centers, and local organizations are well documented [1]. Participation in sports and organized practice also has positive effects on children. Previous studies have shown that children in these environments present better motor coordination, better levels of physical activity, motor competence, and daily task performance when compared to children who are not engaged in these practices [2,3,4,5,6].

There is a positive association between participation in sport in childhood and adolescence and physical activity in adulthood [7]. Therefore, choosing the right path regarding sport participation in childhood is of great importance. Accordingly, two possible models were presented regarding childhood participation in sport: early specialization, and early diversification [8,9,10]. Children who specialize early participate in their chosen sport with a high amount of deliberate practice, and almost no deliberate play in other sports [11]. In the early diversification approach, children participate in various sports [9] and engage in activities designed to promote enjoyment through less structured play.

Physical fitness is nowadays considered one of the most important health markers, as well as a predictor of morbidity and mortality for cardiovascular disease [12]. Physical fitness is, in part, genetically determined, but it can also be greatly influenced by environmental factors. Physical exercise is one of the main determinants [12,13]. Regular participation in moderate and vigorous levels of physical exercise increases physical fitness, which can lead to many health benefits [13]. A low physical fitness status during childhood and adolescence is associated with important health-related outcomes, such as the increased future risk for obesity [14] and cardiovascular diseases [15], impaired skeletal health [16], reduced quality of life [17], and poor mental health [18]. In spite of the healthy benefits of a high level of fitness, children and adolescent‘s performance in fitness tests has declined over the last three decades [19].

Lopes, Stodden, Bianchi, Maia, and Rodrigues (2012) [20], consider motor coordination as one of the most important abilities in early childhood. The aforementioned authors stated that the development of motor coordination should be a key strategy in childhood with an aim to promote long-term obesity prevention and physical activity promotion. Moreover, it has been found that fundamental movement skills are positively associated with health benefits and increased physical activity [21]. Therefore, the early detection and continuous monitoring of children’s motor competence levels is very important. There is a great variation in motor coordination levels within a general population of children. Accordingly, normative standards are important, and so is the determination of possible differences between different kinds of physical activities and their impact on motor competence.

Studies have shown the positive impact of various exercise programs on motor competence in children compared to free play activities or regular curricula in kindergartens or schools [22,23]. In a cross-cultural comparison of motor competence levels (KTK) in children, Bardid, Rudd, Lenoir, Polman, and Barnett (2015) [24] showed that Belgian children generally scored higher on motor competence than Australian children.

Physical testing is used for different purposes and in diverse populations [25,26,27,28]. The importance of testing motor coordination and physical fitness in children was demonstrated earlier [24,29,30]. Moreover, some authors suggest that early success and specialization are not a significant predictor of late success at elite levels across different sports [31,32]. Accordingly, there is a need to investigate the role of different types of physical activity on fitness and motor coordination. We have chosen soccer due to the fact that it is the most popular sport among children, where children specialized probably in the earliest phases of their childhood. Therefore, the purpose of this study was to determine the differences in motor coordination, and physical fitness in children enrolled in soccer and multisport activities.

## 2. Materials and Methods

### 2.1. Subjects

The participants of this study included 147 children, comprised of 88 boys (Age: 7.77 ± 0.56; Height: 126.60 ± 7.1; Weight: 26.5 ± 2.2; BMI: 15.9 ± 1.7), and 59 girls (Age: 7.60 ± 0.8; Height: 127.61 ± 6.4; Weight: 26.52 ± 5.1; BMI: 16.30 ± 1.2) (Table 1). The total sample of subjects was composed of two groups: children who are engaged in organized exercise programs—multisports (*n* = 77, girls = 37)—and children engaged in soccer training (*n* = 70, girls = 24). Children in the current study were required to fulfil the following inclusion criteria: (1) practising in soccer or multisport activity for at least one year; (2) training sessions of at least 60 min and (3) 2 or more days per week of practise; (4) not involved in any other organized sport or activity; (5) participated in more than 80% of sessions during year. Children in soccer clubs exercise 3–4 times a week for 60–90 min. Training consists of specific soccer practice and a relatively large proportion of running and tasks with the ball. The average duration of a child’s training process prior to testing was 15 months of exercising in sports clubs.

Multisport programs consist of indoor exercise programs in a fully equipped gym 2 days per week for ~60 min and one swimming session (60 min). Sessions comprised multiple sports activities and exercises led by educated PE teachers. Each week’s activities were focused on a skill or group of skills from one of the three gross motor skill categories: stability (trunk strength), locomotor (running, hopping, skipping), or manipulation (ball skills). Additionally, children are introduced every week to the most important elements of team and individual sports. Early in each week, children are introduced to motor skills, and movement concepts were added at the end of the week. Later in the program, skill patterns were incorporated into activities.

All participants and their legal guardians provided informed and written consent before participation. Ethics approval was obtained from the University Ethics Board at the Faculty of Sport and Physical Education in Novi Sad (Reference No. 18/2018).

### 2.2. Procedure

Stature and body mass was measured using a stadiometer and a scale according to standardized procedures. Values were recorded to the nearest 0.1 cm and 0.1 kg, respectively.

#### 2.2.1. Motor Coordination

Motor coordination was evaluated with the Kiphard–Schilling body coordination test (Kiphard EJ, Schilling, 1974), Körperkoordination-Testfür-Kinder (KTK), developed and validated on German children. The KTK consists of four subtests: (1) walking backward (WB) three times along each of three balance beams (3 m length; 6, 4.5, and 3 cm width, respectively). A maximum of 24 steps (eight per trial) were counted for each balance beam, which comprised a maximum of 72 steps/points (24 steps, 3 beams) for this test; (2) moving sideways (MS) across the floor in 20 s by stepping from one plate (25 × 25 × 2 cm supported on four legs 3.7 cm high) to the next, transferring to the first plate, stepping on it, and so on. The number of relocations was counted and summed over two trials; (3) hopping for height (HH) on one foot over a foam obstacle of increasing height in consecutive increments of 5 cm. A total of 3, 2, or 1 point(s) were/was awarded for successful performance on the first, second, or third trials, respectively. A maximum of 39 points (ground level + 12 pillows) could be scored for each leg, yielding a possible maximum score of 78; (4) jumping sideways (JS) as fast as possible over a wooden slat (60 × 4 × 2 cm) in 15 s. The number of jumps over two trials was summed. According to the raw scores of these four subtests, an age- and sex-specific percentile rank was calculated using normative data of 1228 normally developing German children [33]. Although the raw scores for each test are commonly transformed using sex- and age-specific tables derived from the original German sample to improve comparability independent of age and sex or converted to an overall motor quotient, the raw score for each test was retained for analysis in the current study.

The “motor quotient” (MQ), a global indicator of MC adjusted for age and gender, was calculated using the four items and used as an indicator of MC. The MQ allows an assessment of the gross motor development in the following categories: ‘severe motor disorder’ (MQ 56–70, percentile 0–2), ‘moderate motor disorder’ (MQ 71–85, percentile 3–16), ‘normal’ (MQ 86–115, percentile 17–84), ‘good’ (MQ 116–130, percentile 85–98) and ‘high’ (MQ 131–145, percentile 99–100).

#### 2.2.2. Physical Fitness

Handgrip strength was measured using a standard adjustable Takei handle analogue handgrip dynamometer (Takei Scientific Instruments Co., Ltd., Niigata, Japan). Children were given a brief demonstration and verbal instructions for the test, and, if necessary, the dynamometer was adjusted according to the child’s hand size. Handgrip strength was measured in a standing position with the shoulder adducted and neutrally rotated, and arms parallel to but not in contact with the body. The participants were asked to squeeze the handle for a maximum of 3–5 s, but no verbal encouragement was given during the test. Two trials were administered for each limb, and the average score was recorded as peak grip strength (kg). Speed-agility was assessed by the 4 × 10 m shuttle run test (SRT). The children had to run and turn at maximum speed between two parallel lines (10 m apart) drawn on the floor, covering a distance of 40 m. To make this easier, two assistants were positioned on both ends, and participants had to touch their hands (placed behind the line) and go back at maximum speed. The best of two attempts were recorded (seconds). A standing long jump was assessed with children standing behind the starting line. They were instructed to push off vigorously and jump as far as possible. They had to land with the feet together and stay upright. The test was repeated twice, and the best score was retained to the nearest 0.1 cm, as the distance between toes at take-off and heels at landing or whichever body part landed nearest to the take-off spot. Cardiorespiratory fitness was assessed using a 20 m shuttle run test. Briefly, children were required to run back and forth on a 20 m course with an audio signal. The test is finished when the child fails to reach the end lines concurrent with the audio signal on two consecutive occasions or when the child stops because of exhaustion. The test results were expressed as the number of laps completed.

### 2.3. Statistical Analysis

Data are expressed as means ± standard deviations. A priori, the G*power 3.1 power analysis software determined the minimum sample size (*n* = 128) given the critical F_(124)_ = 3.918, effect size f = 0.25, *p* = 0.05, 1 − β = 0.8, and number of groups and covariates = 2. Before using parametric tests, the assumption of normality was verified using the Kolmogorov–Smirnov test. While controlling for age and gender effects, differences between the groups (group effect) of children who had been engaged in multisport activities, and the children had enrolled in soccer programs were determined using a one-way ANCOVA. Furthermore, Cohen’s d effect sizes with 95% confidence intervals (95% CI) were used to determine a practically relevant magnitude of difference, which was defined with the following criteria: 0.2–0.5 (small), 0.5–0.79 (moderate), and >0.8 (large) [34]. The level of significance was set at *p* < 0.05. All statistical analyses were performed in SPSS statistical software (SPSS 23.0, IBM Inc., Chicago, IL, USA).

## 3. Results

**Figure 1:**
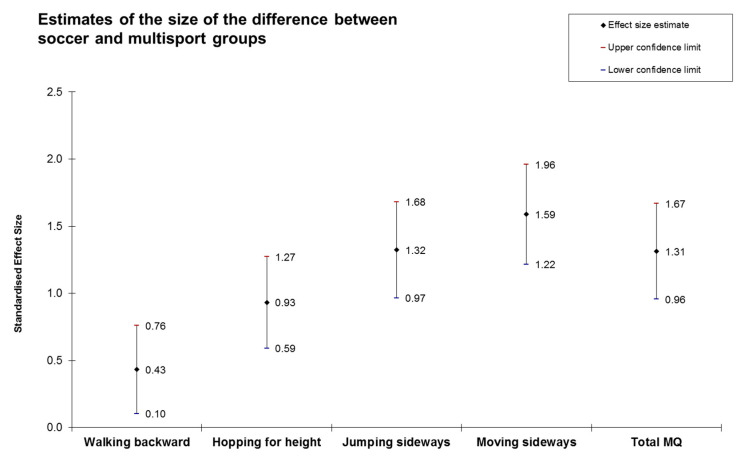
Standardized mean differences and 95% confidence intervals (Cohen’s d ± 95%) between soccer and multisport in gross motor coordination.

Table 2 presents detailed information on the results of the analysis. All variables were normally distributed as suggested by the Kolmogorov–Smirnov test results (*p* > 0.05). After adjusting for age and gender, an ANCOVA showed that the multisport group had a significantly better performance of all motor coordination tests as compared to the soccer group. Large differences between soccer and multisport groups were observed in all motor coordination tests, except in walking backward, where small differences were observed (Figure 1).

**Figure 2:**
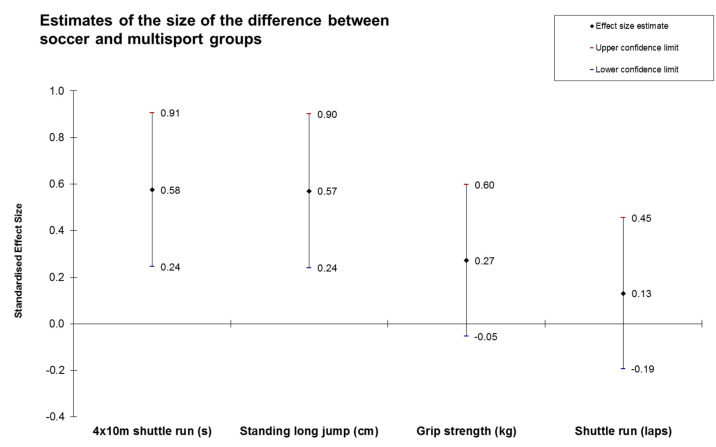
Standardized mean differences and 95% confidence intervals (Cohen’s d ± 95%) between soccer and multisport in 4 × 10 m shuttle run, standing long jump, grip strength, and 20 m shuttle run test.

Children from the soccer group and the multisport group showed similar results for physical fitness without statistically significant differences (*p* > 0.05) after age- and gender-adjustment. However, although nonsignificant, 4 × 10 m shuttle run showed a moderate (d = 0.58) magnitude of difference in favor of the soccer group. On the contrary, standing long jump (d = 0.57) showed moderate differences in favor of the multisport group. For detailed information, Table 3 and Figure 2 are presented.

## 4. Discussion

The purpose of this study was to determine the differences in motor coordination, and physical fitness of children enrolled in soccer and children who enrolled in organized exercise programs—multisports. The main results of this study are that children enrolled in multisport have significantly higher levels of motor coordination than children enrolled in soccer. Higher values in motor coordination tests in children enrolled in a multisport program shows their exposure to the multilateral environment and not only to exercise specialized in soccer. These activities seem to be important and very useful for learning and improving the motor competence of children. Additionally, we found no differences in physical fitness between soccer and multisport activities.

Children’s systematic engagement in a variety of organized physical activities demands high levels of physical effort, neuromuscular coordination, and motor control favoring the development of gross motor coordination and physical fitness [6,35]. Gallahue and Ozmun [36], stated that children develop motor competence through the maturation process, but the best form of motor skills can be achieved with the appropriate training. Moreover, delay in children’s motor performance could occur if children do not learn motor skills or do not have the appropriate amount of activity and exercise [37]. Our study shows similar results: organized physical activity encourages the adequate motor development of children. One more important benefit of well-developed motor coordination is that, when combined with a good level of physical fitness, it can be an important factor for the development of elite athletic performance [2]. Šalaj et al. [38] tried to find out how fundamental movement skills of preschoolers develop depending on different exercise programs. They stated that multisport programs could be recommended as the best form of exercise for preschool children with certain advantages over specific programs of rhythmic gymnastics and soccer. This was confirmed by several studies [2,6,39]. Fransen et al. [2] demonstrated the importance of spending many hours in sports and sampling various sports in the development of gross motor coordination. Another group of authors confirmed that sport-specific training could improve motor coordination characteristics in children but it depends on the sport in which they are enrolled [39]. Regarding gender differences, one interesting study in young tennis players showed that when the children are trained equally, motor coordination is not a significant predictor that could differentiate genders [40]. Additionally, Haugen et al. [41] showed that children with higher levels of motor coordination maintain higher levels of motor coordination and physical fitness throughout childhood and adolescence. The results in the current study showed that the multisport group had better results for motor competence than the children enrolled in soccer. The biggest difference in motor coordination tests was found for jumping sideways (40.18 vs. 57.19) and moving sideways (29.93 vs. 39.31) in favor of the multisport program. Double leg jumps are an integral part of many activities in almost every training session in multisport activities. In general, the emphasis in multisports programs regarding jumping is on long jumps, jumps for a target, jumps over small hurdles, obstacle courses, jumping over lines, and zigzag jumps. The above-mentioned could be one of the reasons for big differences obtained between multisport activities and soccer.

Multisport activities consist of various, and numerous, interesting and complex exercises, in which children develop locomotor and object control skills, and stability, but also improve motor coordination, balance, flexibility, strength, speed, agility, anaerobic endurance, etc. Soccer activities focus on the improvement in physical fitness such as speed, agility, body strength, cardiorespiratory fitness, and ball manipulation. This could be the main reason why we found no statistical difference in physical fitness between these two groups. Both activities showed positive effects on physical fitness regardless of how many hours per week/month/year they practise in soccer or multisport activities.

On the other hand, according to the effect sizes, we found that children in multisport activities performed better in both the standing long jump and handgrip strength, which support the results of Vandorpe et al. [6]. Multisport programs consist of different jumps with both legs in various directions, from different levels of height, in all kinds of usual and unusual body positions, and safe landings from different apparatuses, which is not usual activity in soccer or soccer training. The obstacle course on gymnastic apparatuses, which is a basic organizing method in multisport programs, demands from children movement of their bodies in all directions while hanging on apparatuses using only their hands. The children also have to strongly squeeze all different kind of apparatuses, accessories, and requisites, which positively affects the strength of their hands. Those activities are not usual in soccer activities, so it is not surprising that the multisport group showed better performance in those tests.

Finally, we found better results in the speed/agility test in the group of children enrolled in soccer. This could be explained by the structure of activities performed in soccer training. Speed, change of direction speed and agility are some of the most important body movements in the game of soccer, with or without the ball. Because of this, they are some of the most used exercises in all soccer training, especially at a younger age. Therefore, it can be speculated that the above-mentioned significantly contributed to improvement in agility performance in young soccer players.

We could speculate that both groups would be better than children who do not exercise regularly. Therefore, this could be further examined and considered as a limitation in our study. Physical activity in leisure time could have mediated effects of soccer and multisport activities on physical fitness and motor coordination, hence, the lack of physical activity monitoring in leisure time is another limitation, whose influence must be controlled and evaluated in future studies. López-Gil et al. recently showed that children with normal weight achieved higher results for health-related physical fitness [42], therefore the fact that we did not control the soccer and multisport activities’ effects on physical fitness for weight status could be acknowledged as a limitation as well. Moreover, the lack of information on how long participants have been engaged in soccer or multisport programs is considered a limitation due to the fact that this could affect the results. Finally, the relatively small sample size may be considered a limitation of this study. However, a homogeneous sample in the present study matched according to similar involvement in both activities represents important strength of this study.

## 5. Conclusions

We found that children enrolled in multisport activities have higher levels of motor coordination than children who are enrolled in soccer. Moreover, there were no differences between children in physical fitness. Based on the results of this study, spending time in more than one sport activity might be beneficial in helping to develop motor coordination and also physical fitness in children aged 7–9 years. Therefore, long-term-based and multisport training programs should be considered and encouraged by parents, educators, and other training professionals.

## Figures and Tables

**Table 1:** Sample characteristics.

Characteristics	Soccer	Multisport
Age (years)	7.49 ± 0.90	7.79 ± 0.68
Body height (cm)	125.73 ± 5.85	128.83 ± 5.85
Body mass (kg)	24.11 ± 4.97	27.62 ± 6.72
BMI (weight (kg)/height (m^2^))	15.29 ± 2.31	15.34 ± 2.91

Note: Values are presented as mean ± SD; BMI: body mass index.

**Table 2:** Results of motor coordination tests in children engaged in soccer and multisport.

Motor Coordination Tests	Soccer	Multisport	ANCOVA		
			GROUP F_(1, 144)_	AGE F_(1, 144)_	GENDER F_(1, 144)_
Walking backward	32.21 ± 14.65	38.41 ± 13.93	6.18 *	1.28	0.94
Hopping for height	30.95 ± 10.98	42.27 ± 12.99	30.60 **	3.07	5.04 *
Jumping sideways	40.18 ± 11.16	57.19 ± 14.10	54.77 **	6.84 *	1.32
Moving sideways	29.93 ± 5.76	39.31 ± 5.98	77.82 **	6.1 *	1.69
Total MQ	91.39 ± 16.61	111.86 ± 14.45	58.97 **	4.25 *	6.49 *

Note: Values are presented as mean ± SD; MQ motor quotient; Units for motor coordination tests: Walking backward—number of successful steps; Hopping for height—sum of successful attempts at each height (3 points for the first, 2 points for the second, and 1 point for the third attempt); Jumping sideways—number of correct jumps in 15 s; Moving sideways—number of successful transfers (2 points per transfer) in 20 s; * significant at *p* < 0.05; ** significant at *p* < 0.001.

**Table 3:** Results of physical fitness tests in children engaged in soccer and multisport.

Physical Fitness Tests	Soccer	Multisport	ANCOVA		
			GROUP F_(1, 144)_	AGE F_(1, 144)_	GENDER F_(1, 144)_
4 × 10 m shuttle run (s) ^¥^	13.81 ± 0.88	14.27 ± 0.71	0.01	0.51	0.36
Standing long jump (cm)	118.81 ± 15.01	127.08 ± 13.27	0.62	0.02	1.77
Handgrip strength (kg)	12.43 ± 2.24	13.22 ± 2.89	0.01	0.78	8.01 **
20 m shuttle run (laps)	37.05 ± 9.23	38.22 ± 8.62	1.07	0.00	6.49 *

Note: Values are presented as mean ± SD; ^¥^ reverse scoring; * significant at *p* < 0.05; ** significant at *p* < 0.001.
